# Two cases of venous thromboembolism in siblings after splenectomy due to a novel PROC gene mutation

**DOI:** 10.1186/s12959-024-00597-5

**Published:** 2024-03-19

**Authors:** Yunfang Zhang, Bo Wang, Yuxin Bai, Anxin Wang

**Affiliations:** Department of Pediatric, Shenzhen Second People’s Hospital, Shenzhen University 1st Affiliated Hospital, No.3002, Sungang West Road, Futian District, Shenzhen, Guangdong, Shenzhen, 518019 China

**Keywords:** Venous thromboembolism, Protein C deficiency, Protein C gene, Heterozygous mutation

## Abstract

**Background:**

Venous thromboembolism(VTE)is a common multifactorial disease. Anticoagulant protein deficiency is the most usual hereditary thrombophilia in the Chinese people, which includes protein C(PC), protein S and antithrombin deficiencies.

**Case presentation:**

A retrospective analysis was conducted on clinical manifestations, laboratory tests, genetic information, and other relevant data of siblings diagnosed with VTE in 2020 at the Department of Pediatrics of Shenzhen Second People’s Hospital. The proband, a 12-year-old female, was admitted to the hospital in December 2020 with a complaint of pain in the left lower limb for four days. The examination found that the PC activity was 53%, and B-ultrasound showed bilateral thrombosis of the great saphenous vein in the thigh segment. The proband’s younger brother, a 10-year-old male, was admitted to the hospital in January 2021 due to right lower limb pain for two weeks. PC activity is 40%. B-ultrasound showed superficial venous thrombosis in the left lower limb and upper limb. Both siblings suffered from thalassemia and underwent splenectomy before recurrent thrombosis occurred. The proband’s mother was asymptomatic, and her PC activity was 45%. Both cases were treated with warfarin anticoagulation, and their symptoms improved. The proband’s mother was found to have a heterozygous mutation at this locus through Sanger sequencing.

**Conclusion:**

Protein C deficiency should be considered for venous thromboembolism in childhood. The heterozygous mutation 1204 A > G in PROC exon 9 in this family is reported for the first time.

## Background

VTE is a common multifactorial disease resulting from the interaction of hereditary and environmental risk factors. Anticoagulant protein deficiency is the most usual hereditary thrombophilia in the Chinese people, which includes PC, protein S and antithrombin deficiencies.

PC deficiency is inherited in an autosomal dominant manner. PC is a vital component of the protein C/S anticoagulant system, produced in the liver, and is a 62-kD vitamin K-dependent glycoprotein. Activated PC is naturally produced by PC in the body and has anticoagulant, fibrinolytic, and vascular endothelial barrier stability maintenance properties [1]. When PC is deficient, it leads to the formation of excessive fibrin, resulting in thrombosis. In 1981, Griffin first described this disease, which causes abnormalities in the content or function of PC [2]. We present two cases of siblings who were diagnosed with PC deficiency due to recurrent venous thrombosis. The mutation site of their PROC gene has never been reported before.

## Case presentation

A 12-year-old female patient was admitted to the hospital on December 1, 2020, due to experiencing pain in her left lower limb for four days. Prior to this, between October 5 and October 13, 2020, she had been diagnosed with thrombophlebitis of the great saphenous vein in her right lower limb at another hospital. The patient was prescribed oral warfarin, but it was discontinued after one week due to epistaxis.

Upon admission, the patient had local swelling on the inner left thigh, but no redness or skin temperature abnormalities. The left lower limb had slightly restricted extension, but the patient was able to walk regularly and there were no abnormalities in the right lower limb.

Results of auxiliary examinations indicate normal platelet count, coagulation tests, liver and kidney function, electrolyte levels, serum creatine kinase isoenzyme, and troponin I. However, a B-ultrasound detected abnormal substantial echoes in the thigh segments of both great saphenous veins.

Clinical diagnosis: thrombophlebitis of the superficial veins of both lower limbs, α-thalassemia (--SEA/ααCS), post-splenectomy, siderosis.

The proband’s ten-year-old brother was admitted to the hospital on January 26, 2021, with complaints of pain in his right lower limb for two weeks. Upon physical examination, slight tenderness was observed on the front of the right calf, but there was no redness, swelling, or increase in local skin temperature. The left lower limb appeared normal, though thrombophlebitis was detected in the superficial veins of the left lower and upper limbs. The patient, like the proband, also suffered from α-thalassemia (SEA/ααCS) and siderosis, and had undergone splenectomy.

**Fig. 1:**
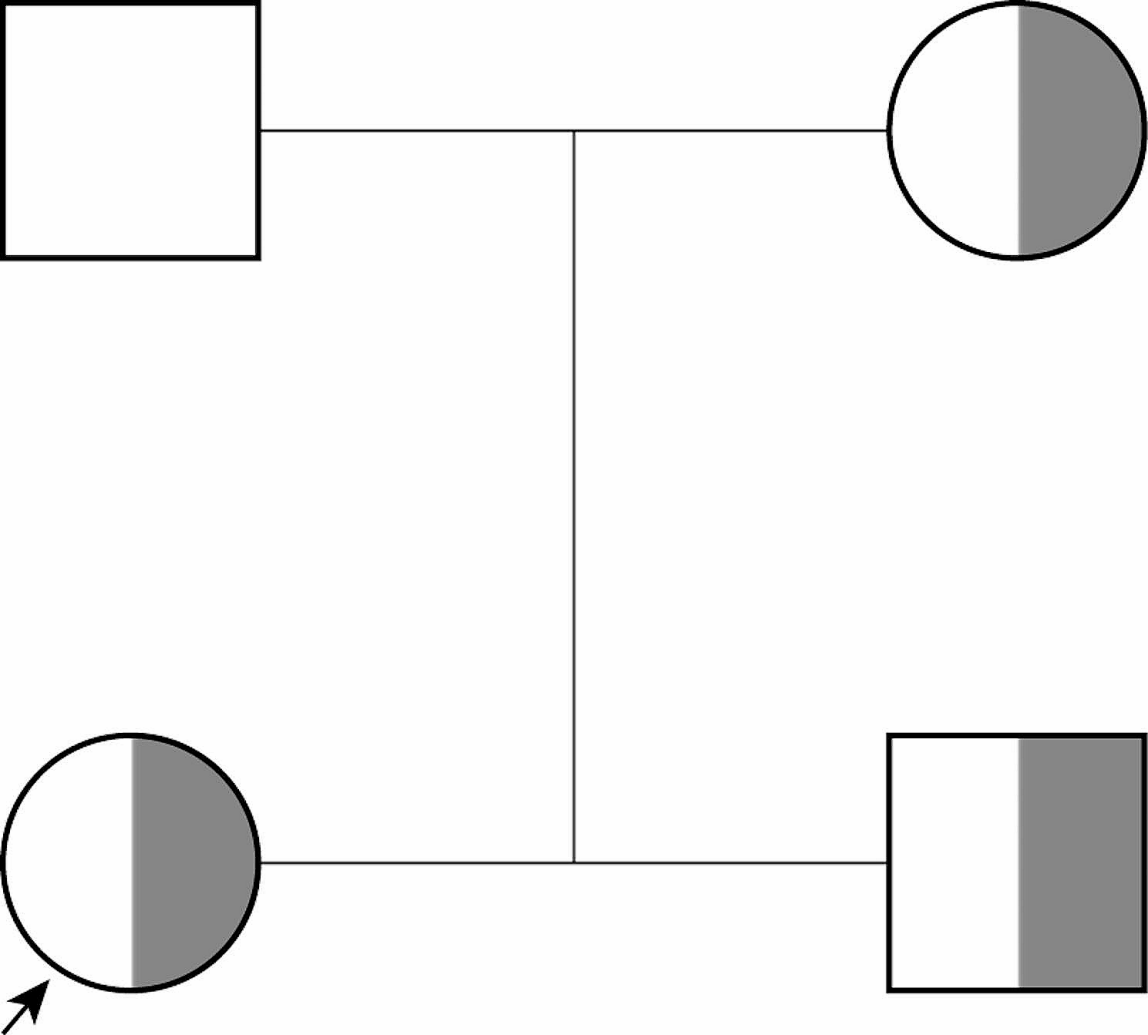
The genealogy of the proband and his family members

The protein C function test was conducted on the proband and their family members. The proband’s protein C activity was found to be 53%, which is below the standard value of 70–130%. The proband’s protein S activity was 72%, within the standard range of 55–123%, and antithrombin activity was 114%, which falls within the standard range of 80–120%. The proband’s brother had a protein C activity of 40%, protein S activity of 75%, and antithrombin activity of 115%. The proband’s mother had a protein C activity of 45%, protein S activity of 74%, and anticoagulant enzyme activity of 100%. You can find the family diagram in Fig. 1.

**Fig. 2:**
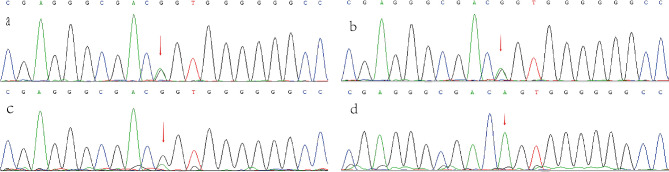
The heterozygous mutation c.1204 A > G in exon 9 of the PROC gene (indicated by the arrow). (a)Proband,(b)Proband’s brother, (c)Proband’s mother, (d)Proband’s father

To proceed with the diagnosis, the parents of the proband were asked to provide informed consent and undergo hemostatic and thrombotic disease panel gene testing. The results revealed that the proband had PROC exon 9 heterozygous mutation, specifically c.1204 A > G, as demonstrated in Fig. 2. Furthermore, Sanger sequencing confirmed that the mutation originated from the mother and that the gene mutation site was the same as that of the proband. According to the guidelines of the American Society of Medical Genetics and Genomics for mutation classification, this gene is classified as likely pathogenic.

Both patients were treated with anticoagulant warfarin to maintain an International normalised ratio (INR) of 1.9–2.5. There were no recurrent venous thromboses or adverse reactions, such as bleeding, during treatment.

## Discussion and conclusions

The PROC gene in humans is situated at 2q13-q14 and covers a length of 11.2 kb. It comprises of 9 exons and is responsible for the production, aggregation, secretion and functioning of protein C (PC). Mutations in the PROC gene commonly cause hereditary PC deficiency. The Human Gene Mutation Database (HGMD) lists 391 (513) PROC gene mutations associated with hereditary protein C deficiency. Majority of these mutations are missense/nonsense mutations with only a few being insertion, deletion and splicing mutations.

Most of the genetic defects causing hereditary PC deficiency are heterozygous. Patients with the heterozygous type usually develop the disease in adulthood and have an increased risk of deep vein thrombosis. However, the symptoms of the homozygous or compound heterozygous type are more severe and often occur in the newborn period. These patients may experience purpura fulminans, pulmonary embolism, and disseminated intravascular coagulation [3]. In this case, genetic analysis of the family revealed that the proband and his brother inherited a heterozygous mutation c.1204 A > G in exon 9 of the PROC gene from their mother.

The genetic variation of the PROC gene c.1204 A > G mutation in a brother and sister was analyzed according to the genetic variation interpretation rules of ACMG/AMP [4] and ClinGen framework. The software predicted a REVEL score of 0.957 points, which indicates that the function was predicted to be harmful. This is considered as strong supporting evidence (PP3). Additionally, this mutation is rare in the general population, which adds supporting evidence (PM2) according to the latest guidelines. Furthermore, the protein activity measurement and clinical phenotype are consistent with PROC gene-related diseases, which adds evidence for PP4. Based on the fact that these three items are met at the same time, the variant site is considered a possible pathogenic variant. It is worth noting that this mutation site is reported for the first time.

The risk of developing VTE due to protein C deficiency varies from person to person, depending on the degree of deficiency and other acquired or inherited factors that increase the risk of blood clots [5]. Studies conducted abroad have shown that the incidence of heterozygous PC deficiency in families with inherited thrombophilia is 6%, while healthy people have a rate of only 0.2% [6]. In China, a study found that around 8% of 202 patients with venous thrombosis had protein C deficiency [7]. Moreover, individuals with protein C deficiency are 10–15 times more likely to develop VTE than those without the deficiency [2].

Compared to healthy individuals, heterozygous individuals have PC levels that are approximately 50% of the standard value. Generally, no clinical symptoms or delayed venous thromboembolism occur [8]. Our patients had PC activities of 53%, 40%, and 45%. The proband and his brother developed superficial vein thrombosis, while the mother was asymptomatic, which is consistent with the literature. Asymptomatic patients with PC deficiency may also be at risk for future VTE, and prospective studies have shown that these patients with heterozygous protein C deficiency have a 2.5% increased risk of thrombosis per year compared to wild-type individuals [9]. The mother needs to be followed up for later thrombosis.

Our study revealed that three family members have PC deficiency. The proband and his brother suffered from venous thrombosis, while the mother did not. The reason for this discrepancy could be attributed to α-thalassemia and splenectomy that the proband and his brother underwent. Patients with thalassemia are vulnerable to VTEs such as deep vein thrombosis, pulmonary embolism, and portal vein thrombosis due to a hypercoagulable state [10]. Several mechanisms lead to hypercoagulability, including chronic platelet activation, changes in RBC membranes, abnormal expression of adhesion molecules on vascular endothelial cells, and dysregulation of hemostasis [10]. Low levels of PC and protein S have been observed in thalassemia patients from different ethnic backgrounds [11]. Regular blood transfusion can reduce the risk of thrombosis, but splenectomy increases it [12]. Acquired risk factors such as pregnancy, exogenous estrogen therapy, immobilization, or surgery have also been shown to increase the incidence of VTE in individuals with PC deficiency [13]. The proband and his brother underwent splenectomies in 2018 and 2019, respectively. This surgery may be a predisposing factor for VTE formation because of procoagulants and abnormal platelets present on the surface of red blood cells [12]. These platelets do not disappear after splenectomy and get removed from circulation, leading to increased PC consumption to control the hypercoagulable state [14–16].

In this study, we discussed a novel mutation in the PROC gene that leads to protein C deficiency and recurrent venous thrombosis after surgery. The mutation was found in a family, and it is the first time this particular mutation site has been reported. Our study adds to the knowledge of the protein C gene’s mutation sites and clinical phenotypes. If a child experiences unexplained venous thromboembolism, protein C deficiency should be considered as a possible cause. It is important to conduct timely genetic testing to confirm the diagnosis.

## Data Availability

No datasets were generated or analysed during the current study.

## Abbreviations

PC: Protein C
VTE: Venous thromboembolism
